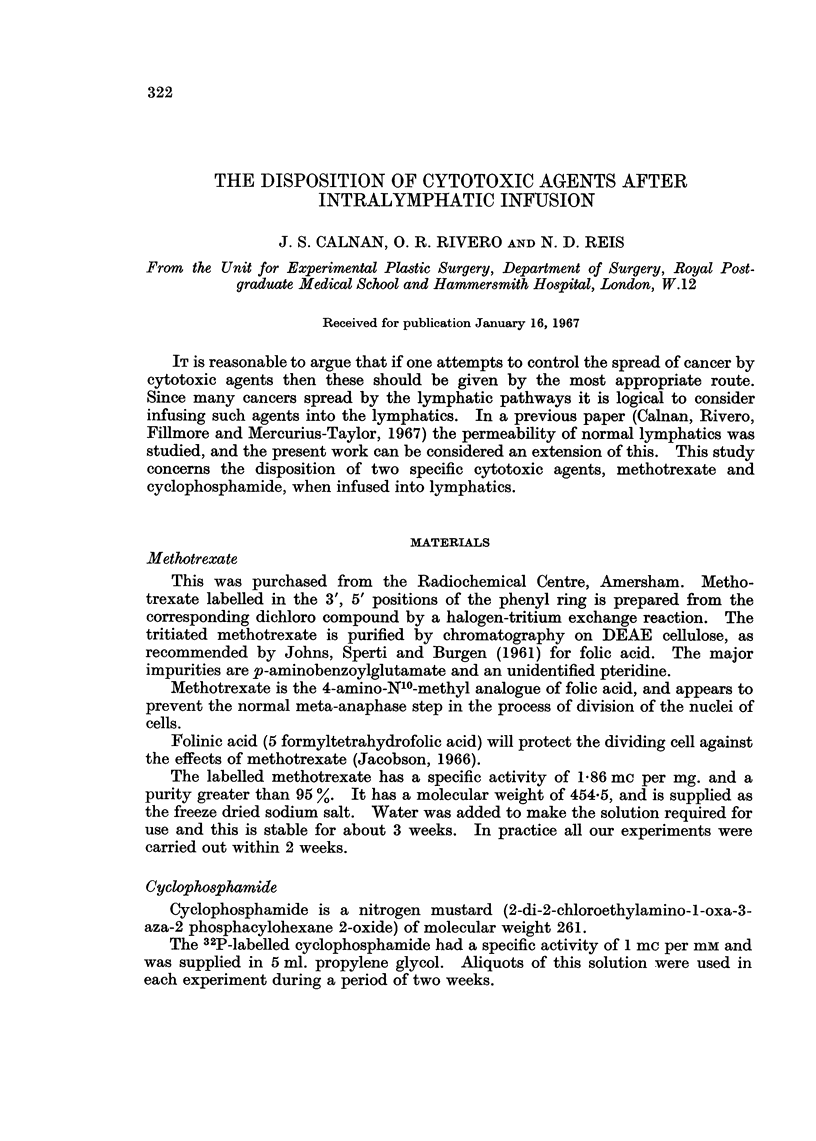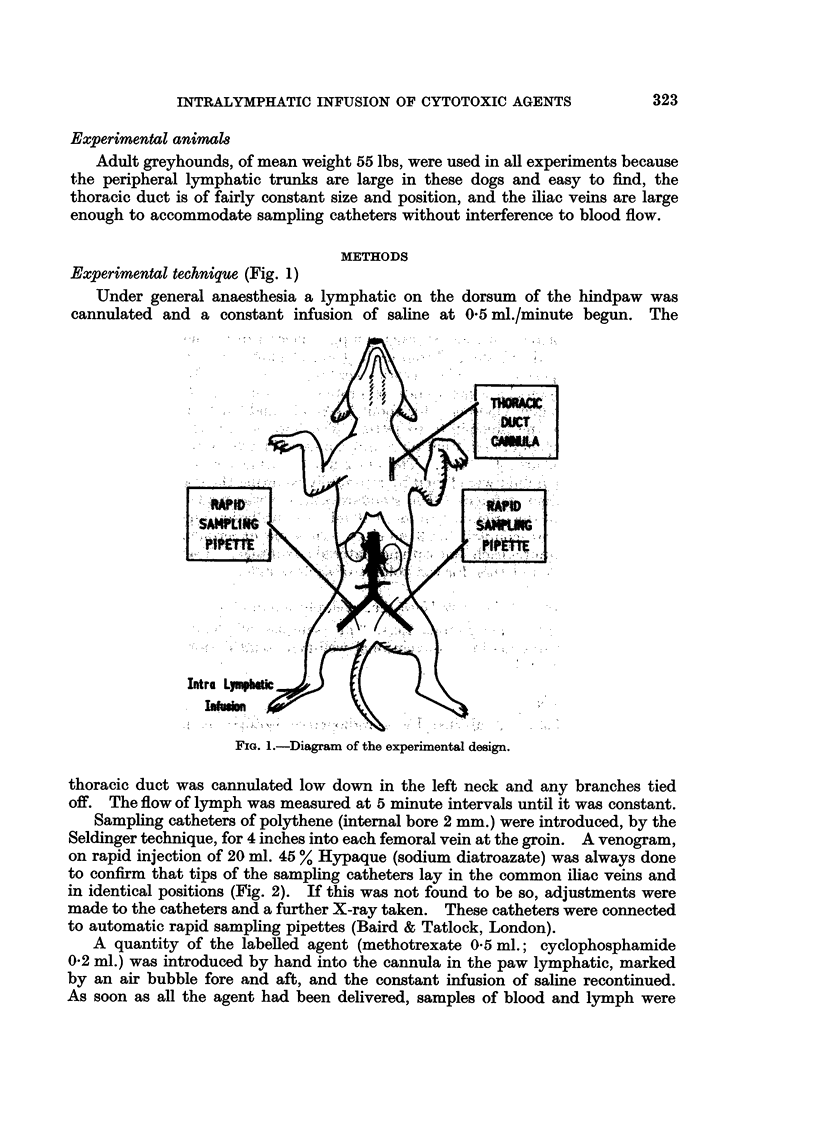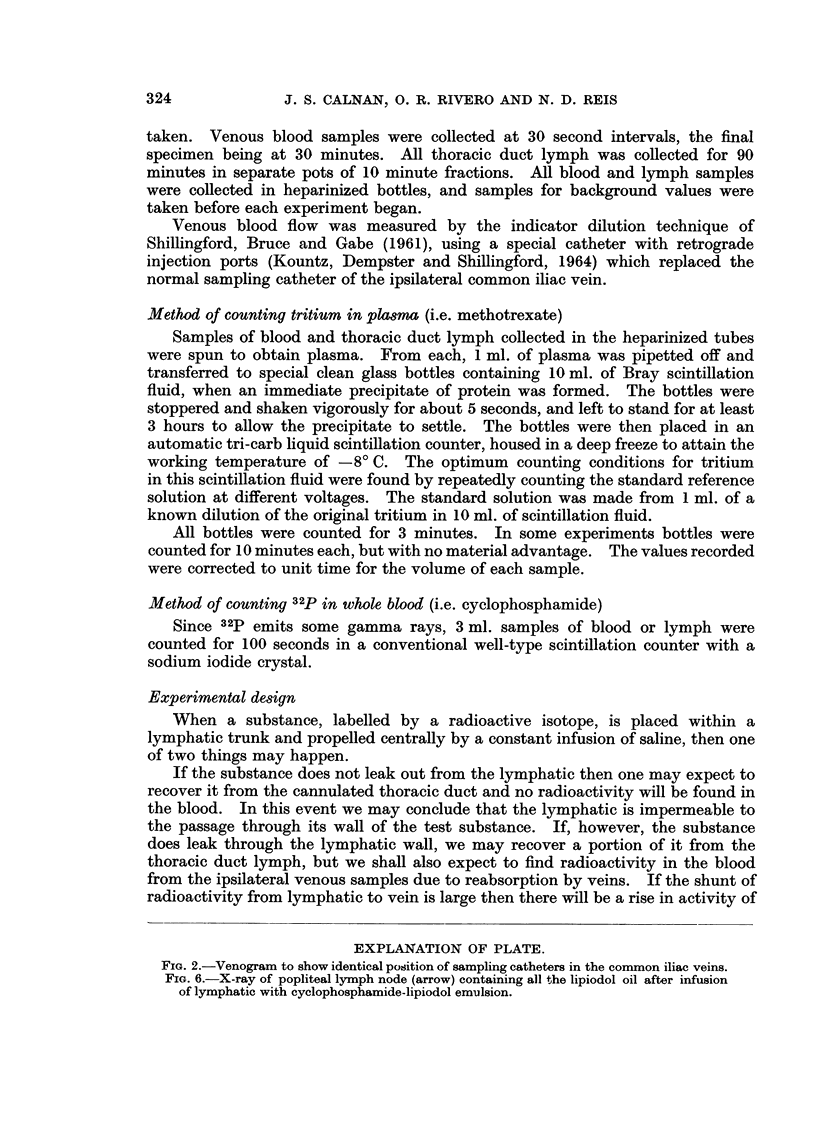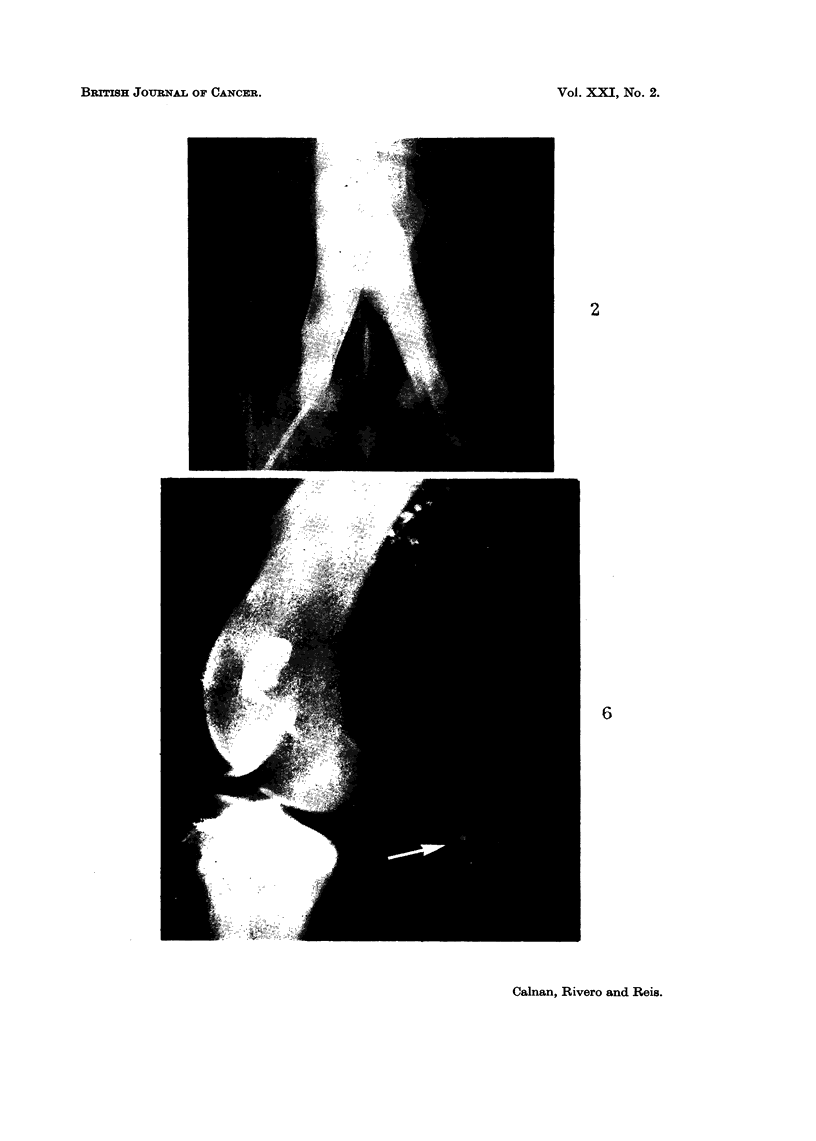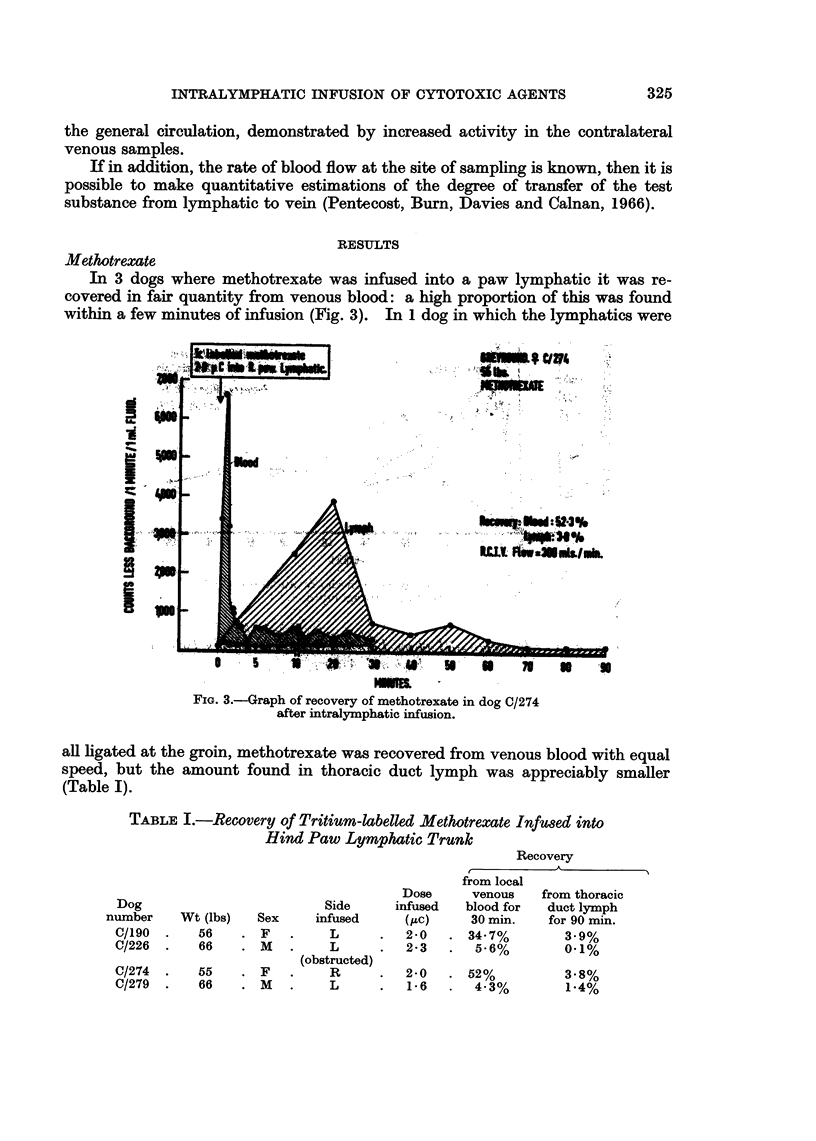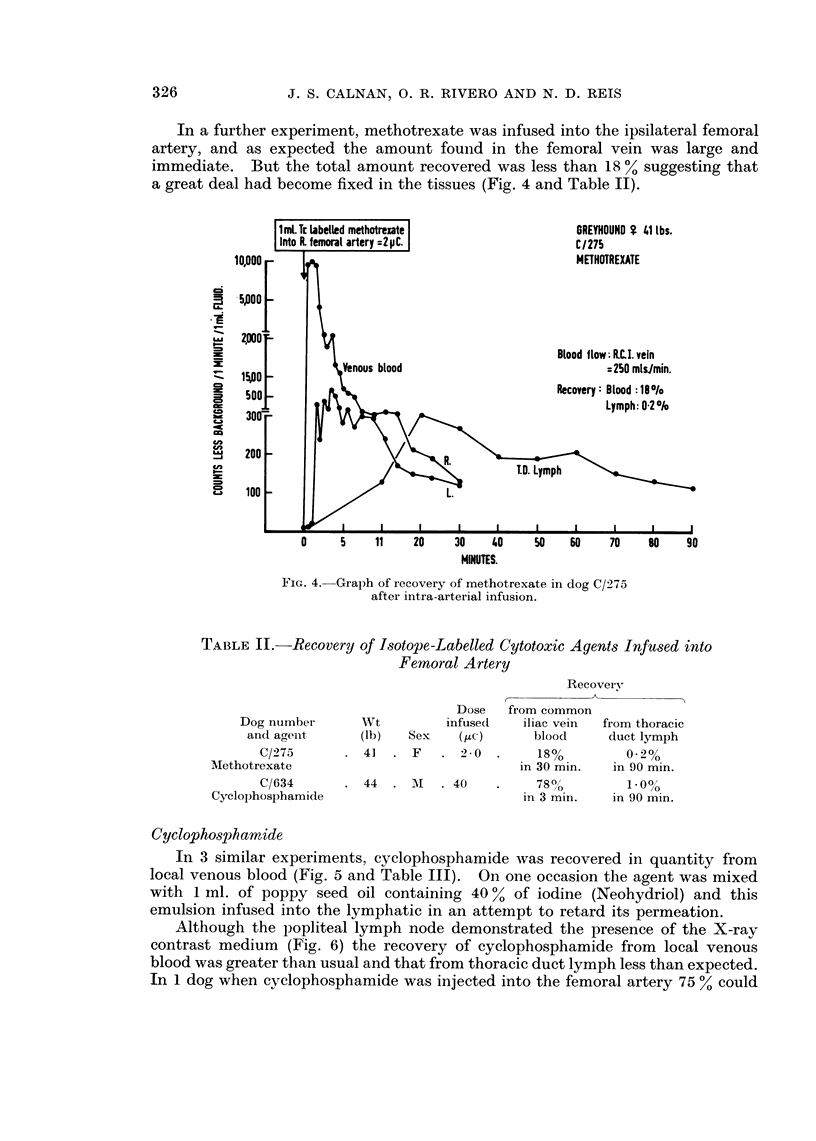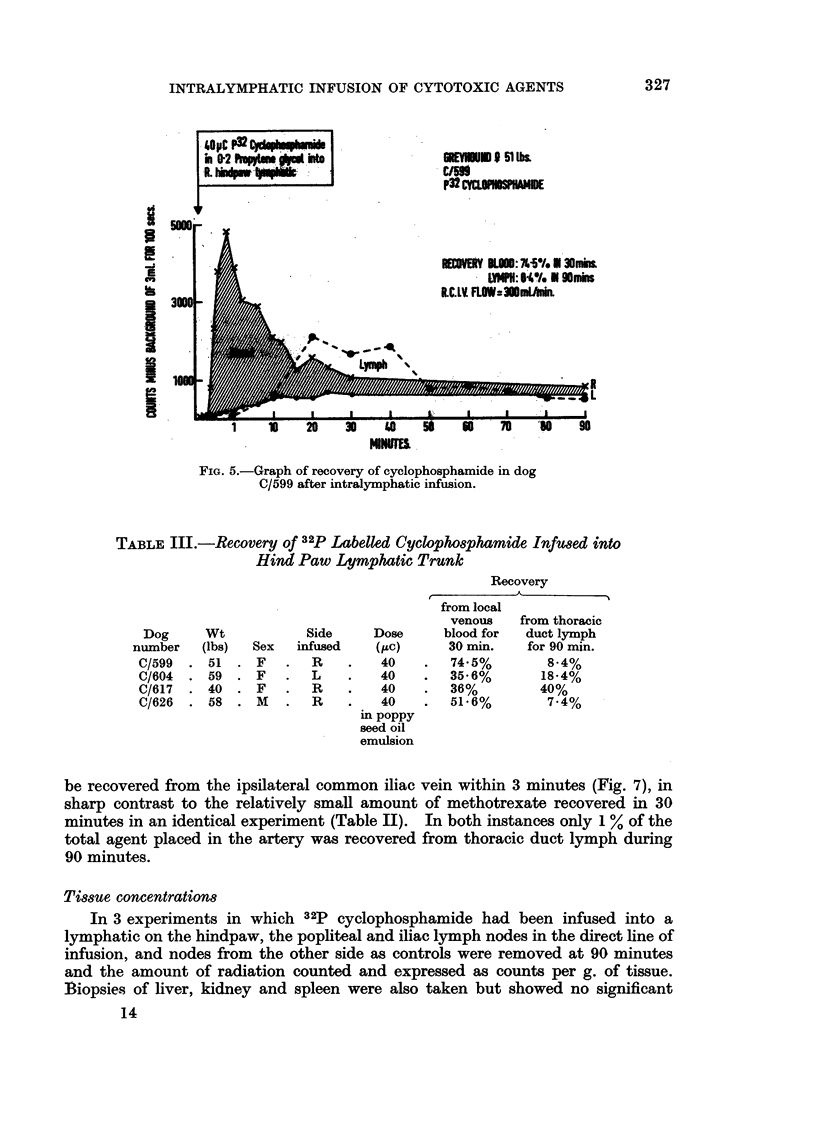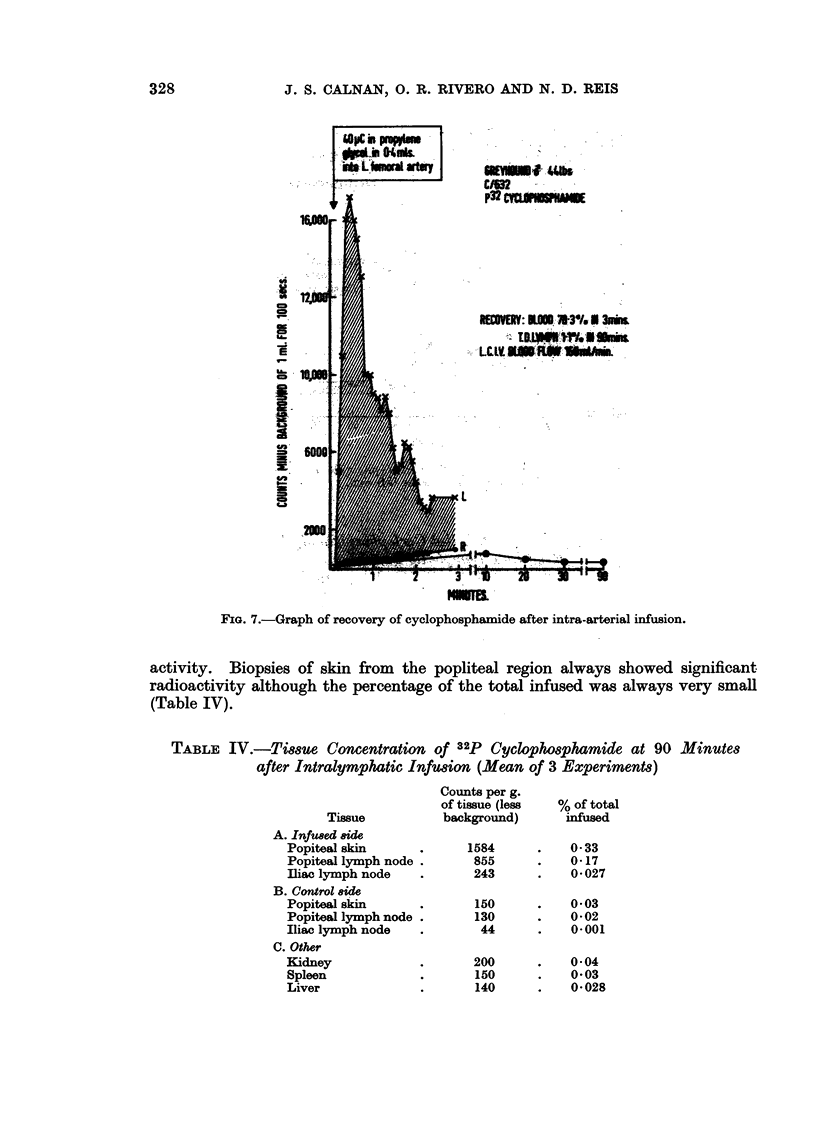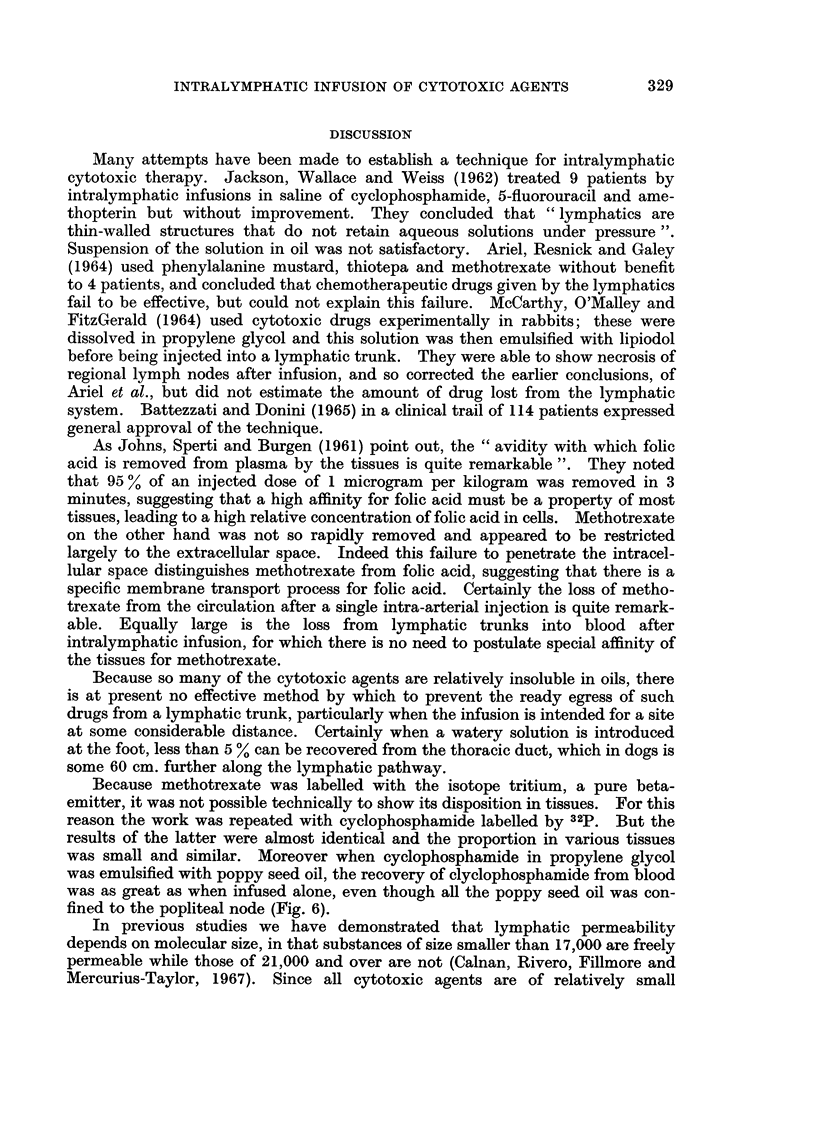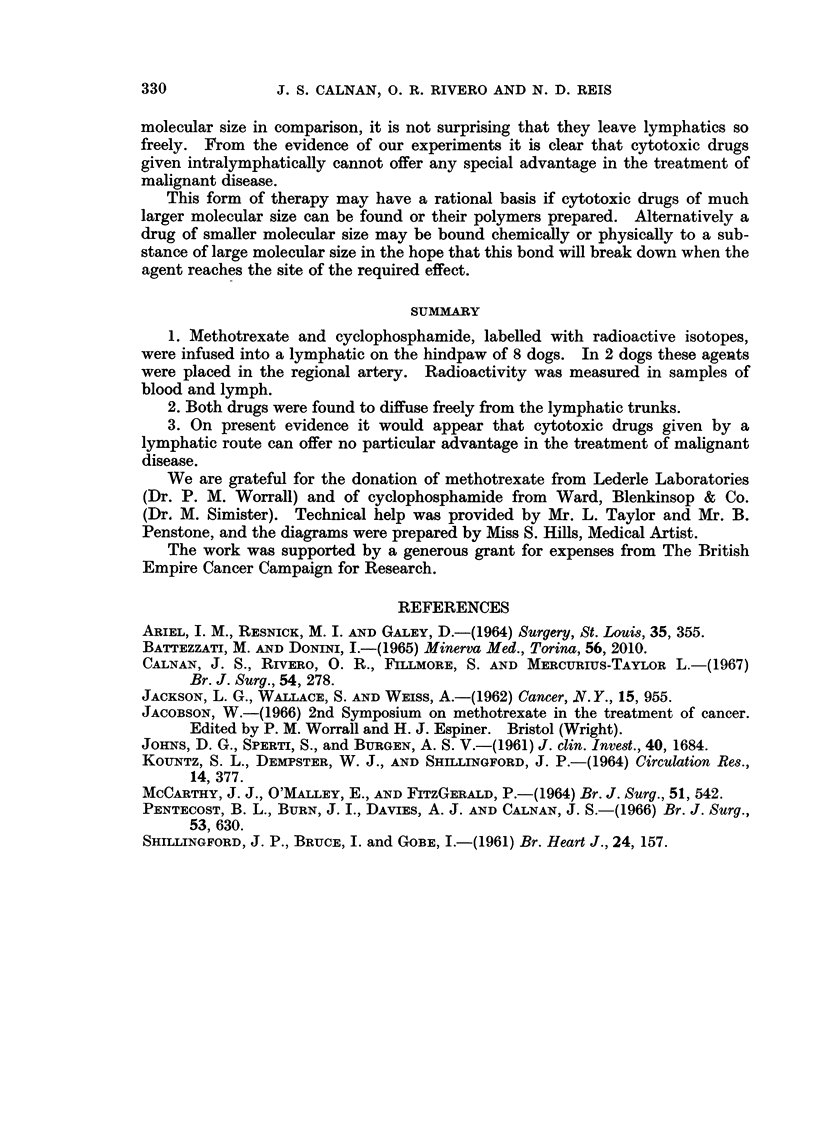# The disposition of cytotoxic agents after intralymphatic infusion.

**DOI:** 10.1038/bjc.1967.35

**Published:** 1967-06

**Authors:** J. S. Calnan, O. R. Rivero, N. D. Reis

## Abstract

**Images:**


### THE DISPOSITION OF CYTOTOXIC AGENTS AFTER INTRALYMPHATIC INFUSION

J. S. CALNAN, O. R. RIVERO AND N. D. REIS

*From the Unit for Experimental Plastic Surgery, Department of Surgery, Royal Post-graduate Medical School and Hammersmith Hospital, London, W.12*

Received for publication January 16, 1967

IT is reasonable to argue that if one attempts to control the spread of cancer by cytotoxic agents then these should be given by the most appropriate route. Since many cancers spread by the lymphatic pathways it is logical to consider infusing such agents into the lymphatics. In a previous paper (Calnan, Rivero, Fillmore and Mercurius-Taylor, 1967) the permeability of normal lymphatics was studied, and the present work can be considered an extension of this. This study concerns the disposition of two specific cytotoxic agents, methotrexate and cyclophosphamide, when infused into lymphatics.

### MATERIALS

### *Methotrexate*

This was purchased from the Radiochemical Centre, Amersham. Methotrexate labelled in the 3′, 5′ positions of the phenyl ring is prepared from the corresponding dichloro compound by a halogen-tritium exchange reaction. The tritiated methotrexate is purified by chromatography on DEAE cellulose, as recommended by Johns, Sperti and Burgen (1961) for folic acid. The major impurities are *p*-aminobenzoylglutamate and an unidentified pteridine.

Methotrexate is the 4-amino-$N^{10}$-methyl analogue of folic acid, and appears to prevent the normal meta-anaphase step in the process of division of the nuclei of cells.

Folinic acid (5 formyltetrahydrofolic acid) will protect the dividing cell against the effects of methotrexate (Jacobson, 1966).

The labelled methotrexate has a specific activity of 1·86 mc per mg. and a purity greater than 95 %. It has a molecular weight of 454·5, and is supplied as the freeze dried sodium salt. Water was added to make the solution required for use and this is stable for about 3 weeks. In practice all our experiments were carried out within 2 weeks.

### *Cyclophosphamide*

Cyclophosphamide is a nitrogen mustard (2-di-2-chloroethylamino-1-oxa-3-aza-2 phosphacylohexane 2-oxide) of molecular weight 261.

The $^{32}P$-labelled cyclophosphamide had a specific activity of 1 mc per mM and was supplied in 5 ml. propylene glycol. Aliquots of this solution were used in each experiment during a period of two weeks.

*Experimental animals*

Adult greyhounds, of mean weight 55 lbs, were used in all experiments because the peripheral lymphatic trunks are large in these dogs and easy to find, the thoracic duct is of fairly constant size and position, and the iliac veins are large enough to accommodate sampling catheters without interference to blood flow.

### METHODS

*Experimental technique* (Fig. 1)

Under general anaesthesia a lymphatic on the dorsum of the hindpaw was cannulated and a constant infusion of saline at 0·5 ml./minute begun. The

FIG. 1.—Diagram of the experimental design.

thoracic duct was cannulated low down in the left neck and any branches tied off. The flow of lymph was measured at 5 minute intervals until it was constant.

Sampling catheters of polythene (internal bore 2 mm.) were introduced, by the Seldinger technique, for 4 inches into each femoral vein at the groin. A venogram, on rapid injection of 20 ml. 45 % Hypaque (sodium diatroazate) was always done to confirm that tips of the sampling catheters lay in the common iliac veins and in identical positions (Fig. 2). If this was not found to be so, adjustments were made to the catheters and a further X-ray taken. These catheters were connected to automatic rapid sampling pipettes (Baird & Tatlock, London).

A quantity of the labelled agent (methotrexate 0·5 ml.; cyclophosphamide 0·2 ml.) was introduced by hand into the cannula in the paw lymphatic, marked by an air bubble fore and aft, and the constant infusion of saline recontinued. As soon as all the agent had been delivered, samples of blood and lymph were taken. Venous blood samples were collected at 30 second intervals, the final specimen being at 30 minutes. All thoracic duct lymph was collected for 90 minutes in separate pots of 10 minute fractions. All blood and lymph samples were collected in heparinized bottles, and samples for background values were taken before each experiment began.

Venous blood flow was measured by the indicator dilution technique of Shillingford, Bruce and Gabe (1961), using a special catheter with retrograde injection ports (Kountz, Dempster and Shillingford, 1964) which replaced the normal sampling catheter of the ipsilateral common iliac vein.

*Method of counting tritium in plasma* (i.e. methotrexate)

Samples of blood and thoracic duct lymph collected in the heparinized tubes were spun to obtain plasma. From each, 1 ml. of plasma was pipetted off and transferred to special clean glass bottles containing 10 ml. of Bray scintillation fluid, when an immediate precipitate of protein was formed. The bottles were stoppered and shaken vigorously for about 5 seconds, and left to stand for at least 3 hours to allow the precipitate to settle. The bottles were then placed in an automatic tri-carb liquid scintillation counter, housed in a deep freeze to attain the working temperature of $-8°$ C. The optimum counting conditions for tritium in this scintillation fluid were found by repeatedly counting the standard reference solution at different voltages. The standard solution was made from 1 ml. of a known dilution of the original tritium in 10 ml. of scintillation fluid.

All bottles were counted for 3 minutes. In some experiments bottles were counted for 10 minutes each, but with no material advantage. The values recorded were corrected to unit time for the volume of each sample.

*Method of counting $^{32}P$ in whole blood* (i.e. cyclophosphamide)

Since $^{32}$P emits some gamma rays, 3 ml. samples of blood or lymph were counted for 100 seconds in a conventional well-type scintillation counter with a sodium iodide crystal.

*Experimental design*

When a substance, labelled by a radioactive isotope, is placed within a lymphatic trunk and propelled centrally by a constant infusion of saline, then one of two things may happen.

If the substance does not leak out from the lymphatic then one may expect to recover it from the cannulated thoracic duct and no radioactivity will be found in the blood. In this event we may conclude that the lymphatic is impermeable to the passage through its wall of the test substance. If, however, the substance does leak through the lymphatic wall, we may recover a portion of it from the thoracic duct lymph, but we shall also expect to find radioactivity in the blood from the ipsilateral venous samples due to reabsorption by veins. If the shunt of radioactivity from lymphatic to vein is large then there will be a rise in activity of

---

EXPLANATION OF PLATE.

FIG. 2.—Venogram to show identical position of sampling catheters in the common iliac veins.

FIG. 6.—X-ray of popliteal lymph node (arrow) containing all the lipiodol oil after infusion of lymphatic with cyclophosphamide-lipiodol emulsion.

2

6

Calnan, Rivero and Reis.

the general circulation, demonstrated by increased activity in the contralateral venous samples.

If in addition, the rate of blood flow at the site of sampling is known, then it is possible to make quantitative estimations of the degree of transfer of the test substance from lymphatic to vein (Pentecost, Burn, Davies and Calnan, 1966).

### RESULTS

*Methotrexate*

In 3 dogs where methotrexate was infused into a paw lymphatic it was recovered in fair quantity from venous blood: a high proportion of this was found within a few minutes of infusion (Fig. 3). In 1 dog in which the lymphatics were

FIG. 3.—Graph of recovery of methotrexate in dog C/274 after intralymphatic infusion.

all ligated at the groin, methotrexate was recovered from venous blood with equal speed, but the amount found in thoracic duct lymph was appreciably smaller (Table I).

TABLE I.—*Recovery of Tritium-labelled Methotrexate Infused into Hind Paw Lymphatic Trunk*

| Dog number | Wt (lbs) | Sex | Side infused | Dose infused (μc) | Recovery: from local venous blood for 30 min. | Recovery: from thoracic duct lymph for 90 min. |
|---|---|---|---|---|---|---|
| C/190 | 56 | F | L | 2·0 | 34·7% | 3·9% |
| C/226 | 66 | M | L (obstructed) | 2·3 | 5·6% | 0·1% |
| C/274 | 55 | F | R | 2·0 | 52% | 3·8% |
| C/279 | 66 | M | L | 1·6 | 4·3% | 1·4% |

In a further experiment, methotrexate was infused into the ipsilateral femoral artery, and as expected the amount found in the femoral vein was large and immediate. But the total amount recovered was less than 18 % suggesting that a great deal had become fixed in the tissues (Fig. 4 and Table II).

FIG. 4.—Graph of recovery of methotrexate in dog C/275 after intra-arterial infusion.

TABLE II.—*Recovery of Isotope-Labelled Cytotoxic Agents Infused into Femoral Artery*

| | | | | Recovery | |
|---|---|---|---|---|---|
| Dog number and agent | Wt (lb) | Sex | Dose infused (μc) | from common iliac vein blood | from thoracic duct lymph |
| C/275 Methotrexate | 41 | F | 2·0 | 18% in 30 min. | 0·2% in 90 min. |
| C/634 Cyclophosphamide | 44 | M | 40 | 78% in 3 min. | 1·0% in 90 min. |

*Cyclophosphamide*

In 3 similar experiments, cyclophosphamide was recovered in quantity from local venous blood (Fig. 5 and Table III). On one occasion the agent was mixed with 1 ml. of poppy seed oil containing 40 % of iodine (Neohydriol) and this emulsion infused into the lymphatic in an attempt to retard its permeation.

Although the popliteal lymph node demonstrated the presence of the X-ray contrast medium (Fig. 6) the recovery of cyclophosphamide from local venous blood was greater than usual and that from thoracic duct lymph less than expected. In 1 dog when cyclophosphamide was injected into the femoral artery 75 % could

FIG. 5.—Graph of recovery of cyclophosphamide in dog C/599 after intralymphatic infusion.

TABLE III.—*Recovery of* $^{32}P$ *Labelled Cyclophosphamide Infused into Hind Paw Lymphatic Trunk*

| Dog number | Wt (lbs) | Sex | Side infused | Dose (μc) | Recovery: from local venous blood for 30 min. | Recovery: from thoracic duct lymph for 90 min. |
|---|---|---|---|---|---|---|
| C/599 | 51 | F | R | 40 | 74·5% | 8·4% |
| C/604 | 59 | F | L | 40 | 35·6% | 18·4% |
| C/617 | 40 | F | R | 40 | 36% | 40% |
| C/626 | 58 | M | R | 40 in poppy seed oil emulsion | 51·6% | 7·4% |

be recovered from the ipsilateral common iliac vein within 3 minutes (Fig. 7), in sharp contrast to the relatively small amount of methotrexate recovered in 30 minutes in an identical experiment (Table II). In both instances only 1 % of the total agent placed in the artery was recovered from thoracic duct lymph during 90 minutes.

*Tissue concentrations*

In 3 experiments in which $^{32}P$ cyclophosphamide had been infused into a lymphatic on the hindpaw, the popliteal and iliac lymph nodes in the direct line of infusion, and nodes from the other side as controls were removed at 90 minutes and the amount of radiation counted and expressed as counts per g. of tissue. Biopsies of liver, kidney and spleen were also taken but showed no significant

FIG. 7.—Graph of recovery of cyclophosphamide after intra-arterial infusion.

activity. Biopsies of skin from the popliteal region always showed significant radioactivity although the percentage of the total infused was always very small (Table IV).

TABLE IV.—*Tissue Concentration of* $^{32}P$ *Cyclophosphamide at* 90 *Minutes after Intralymphatic Infusion* (*Mean of* 3 *Experiments*)

| Tissue | Counts per g. of tissue (less background) | % of total infused |
|---|---|---|
| A. *Infused side* | | |
| Popiteal skin | 1584 | 0·33 |
| Popiteal lymph node | 855 | 0·17 |
| Iliac lymph node | 243 | 0·027 |
| B. *Control side* | | |
| Popiteal skin | 150 | 0·03 |
| Popiteal lymph node | 130 | 0·02 |
| Iliac lymph node | 44 | 0·001 |
| C. *Other* | | |
| Kidney | 200 | 0·04 |
| Spleen | 150 | 0·03 |
| Liver | 140 | 0·028 |

### DISCUSSION

Many attempts have been made to establish a technique for intralymphatic cytotoxic therapy. Jackson, Wallace and Weiss (1962) treated 9 patients by intralymphatic infusions in saline of cyclophosphamide, 5-fluorouracil and amethopterin but without improvement. They concluded that " lymphatics are thin-walled structures that do not retain aqueous solutions under pressure ". Suspension of the solution in oil was not satisfactory. Ariel, Resnick and Galey (1964) used phenylalanine mustard, thiotepa and methotrexate without benefit to 4 patients, and concluded that chemotherapeutic drugs given by the lymphatics fail to be effective, but could not explain this failure. McCarthy, O'Malley and FitzGerald (1964) used cytotoxic drugs experimentally in rabbits; these were dissolved in propylene glycol and this solution was then emulsified with lipiodol before being injected into a lymphatic trunk. They were able to show necrosis of regional lymph nodes after infusion, and so corrected the earlier conclusions, of Ariel *et al.*, but did not estimate the amount of drug lost from the lymphatic system. Battezzati and Donini (1965) in a clinical trail of 114 patients expressed general approval of the technique.

As Johns, Sperti and Burgen (1961) point out, the " avidity with which folic acid is removed from plasma by the tissues is quite remarkable ". They noted that 95% of an injected dose of 1 microgram per kilogram was removed in 3 minutes, suggesting that a high affinity for folic acid must be a property of most tissues, leading to a high relative concentration of folic acid in cells. Methotrexate on the other hand was not so rapidly removed and appeared to be restricted largely to the extracellular space. Indeed this failure to penetrate the intracellular space distinguishes methotrexate from folic acid, suggesting that there is a specific membrane transport process for folic acid. Certainly the loss of methotrexate from the circulation after a single intra-arterial injection is quite remarkable. Equally large is the loss from lymphatic trunks into blood after intralymphatic infusion, for which there is no need to postulate special affinity of the tissues for methotrexate.

Because so many of the cytotoxic agents are relatively insoluble in oils, there is at present no effective method by which to prevent the ready egress of such drugs from a lymphatic trunk, particularly when the infusion is intended for a site at some considerable distance. Certainly when a watery solution is introduced at the foot, less than 5 % can be recovered from the thoracic duct, which in dogs is some 60 cm. further along the lymphatic pathway.

Because methotrexate was labelled with the isotope tritium, a pure beta-emitter, it was not possible technically to show its disposition in tissues. For this reason the work was repeated with cyclophosphamide labelled by $^{32}P$. But the results of the latter were almost identical and the proportion in various tissues was small and similar. Moreover when cyclophosphamide in propylene glycol was emulsified with poppy seed oil, the recovery of clyclophosphamide from blood was as great as when infused alone, even though all the poppy seed oil was confined to the popliteal node (Fig. 6).

In previous studies we have demonstrated that lymphatic permeability depends on molecular size, in that substances of size smaller than 17,000 are freely permeable while those of 21,000 and over are not (Calnan, Rivero, Fillmore and Mercurius-Taylor, 1967). Since all cytotoxic agents are of relatively small molecular size in comparison, it is not surprising that they leave lymphatics so freely. From the evidence of our experiments it is clear that cytotoxic drugs given intralymphatically cannot offer any special advantage in the treatment of malignant disease.

This form of therapy may have a rational basis if cytotoxic drugs of much larger molecular size can be found or their polymers prepared. Alternatively a drug of smaller molecular size may be bound chemically or physically to a substance of large molecular size in the hope that this bond will break down when the agent reaches the site of the required effect.

### SUMMARY

1. Methotrexate and cyclophosphamide, labelled with radioactive isotopes, were infused into a lymphatic on the hindpaw of 8 dogs. In 2 dogs these agents were placed in the regional artery. Radioactivity was measured in samples of blood and lymph.

2. Both drugs were found to diffuse freely from the lymphatic trunks.

3. On present evidence it would appear that cytotoxic drugs given by a lymphatic route can offer no particular advantage in the treatment of malignant disease.

We are grateful for the donation of methotrexate from Lederle Laboratories (Dr. P. M. Worrall) and of cyclophosphamide from Ward, Blenkinsop & Co. (Dr. M. Simister). Technical help was provided by Mr. L. Taylor and Mr. B. Penstone, and the diagrams were prepared by Miss S. Hills, Medical Artist.

The work was supported by a generous grant for expenses from The British Empire Cancer Campaign for Research.

### REFERENCES

Ariel, I. M., Resnick, M. I. and Galey, D.—(1964) *Surgery, St. Louis*, **35**, 355.

Battezzati, M. and Donini, I.—(1965) *Minerva Med., Torina*, **56**, 2010.

Calnan, J. S., Rivero, O. R., Fillmore, S. and Mercurius-Taylor L.—(1967) *Br. J. Surg.*, **54**, 278.

Jackson, L. G., Wallace, S. and Weiss, A.—(1962) *Cancer, N.Y.*, **15**, 955.

Jacobson, W.—(1966) 2nd Symposium on methotrexate in the treatment of cancer. Edited by P. M. Worrall and H. J. Espiner. Bristol (Wright).

Johns, D. G., Sperti, S., and Burgen, A. S. V.—(1961) *J. clin. Invest.*, **40**, 1684.

Kountz, S. L., Dempster, W. J., and Shillingford, J. P.—(1964) *Circulation Res.*, **14**, 377.

McCarthy, J. J., O'Malley, E., and FitzGerald, P.—(1964) *Br. J. Surg.*, **51**, 542.

Pentecost, B. L., Burn, J. I., Davies, A. J. and Calnan, J. S.—(1966) *Br. J. Surg.*, **53**, 630.

Shillingford, J. P., Bruce, I. and Gobe, I.—(1961) *Br. Heart J.*, **24**, 157.